# Emerging role of microbiota derived outer membrane vesicles to preventive, therapeutic and diagnostic proposes

**DOI:** 10.1186/s13027-023-00480-4

**Published:** 2023-01-19

**Authors:** Saba Jalalifar, Hassan Morovati Khamsi, Seyed Reza Hosseini-Fard, Sajad Karampoor, Bahar Bajelan, Gholamreza Irajian, Rasoul Mirzaei

**Affiliations:** 1 Microbial Biotechnology Research Center, Iran University of Medical Sciences, Tehran, Iran; 2 Department of Microbiology, School of Medicine, Iran University of Medical Sciences, Tehran, Iran; 3 Department of Quality Control, Razi Vaccine and Serum Research Institute, Agricultural Research, Education and Extension Organization (AREEO), Karaj, Iran; 4 Department of Biochemistry, School of Medicine, Tehran University of Medical Sciences, Tehran, Iran; 5 Gastrointestinal and Liver Diseases Research Center, Iran University of Medical Sciences, Tehran, Iran; 6 School of Medicine, Alborz University of Medical Sciences, Karaj, Iran; 7 Venom and Biotherapeutics Molecules Lab, Medical Biotechnology Department, Biotechnology Research Center, Pasteur Institute of Iran, Tehran, Iran

**Keywords:** Microbiota, Outer membrane vesicle, Vaccine, Adjuvant, Drug delivery, Biomarker

## Abstract

The role of gut microbiota and its products in human health and disease is profoundly investigated. The communication between gut microbiota and the host involves a complicated network of signaling pathways via biologically active molecules generated by intestinal microbiota. Some of these molecules could be assembled within nanoparticles known as outer membrane vesicles (OMVs). Recent studies propose that OMVs play a critical role in shaping immune responses, including homeostasis and acute inflammatory responses. Moreover, these OMVs have an immense capacity to be applied in medical research, such as OMV-based vaccines and drug delivery. This review presents a comprehensive overview of emerging knowledge about biogenesis, the role, and application of these bacterial-derived OMVs, including OMV-based vaccines, OMV adjuvants characteristics, OMV vehicles (in conjugated vaccines), cancer immunotherapy, and drug carriers and delivery systems. Moreover, we also highlight the significance of the potential role of these OMVs in diagnosis and therapy.

## Key points

- OMVs are nanosized proteoliposomes derived from the outer membrane of Gram-negative bacteria.
- Based on the physiological characteristics of OMVs, The delivery of therapeutic cargos, such as miRNAs and proteins to tissues, has now been identified.
- Also, Designing powerful nanocarriers has administered bioengineering to target particular delivery of therapeutics for OMVs

## Introduction

Gut microbiota plays a crucial role in the absorption of minerals and nutrients, synthesizing enzymes, vitamins, amino acids, and modulating the immune system [1–3]. Besides, a growing body of evidence shows that bacterial dysbiosis contributes to the development of some disorders, such as inflammatory bowel disease (IBD), obesity, irritable bowel syndrome (IBS), diabetes, cancer, multiple sclerosis (MS), and neurological diseases [4, 5]. On the other hand, the interplay between gut microbiota and immune cells is involved in the homeostasis of the gastrointestinal (GI) tract, health maintenance, and infection prevention in the host [6–8].

The shedding process of membrane vesicles (MVs) has been characterized as an evolutionarily conserved mechanism across eukaryotes and prokaryotes for intercellular communications [9]. These nano-sized, spherical, and bilayer proteolipid extracellular MVs harbor subsets of lipids, proteins, nucleic acids, as well as metabolites [9]. According to the hosts that extracellular vesicles (EVs) are derived from, these molecules are differently named, such as outer MVs (OMVs) for Gram-negative microorganisms; MVs for Gram-positive microorganisms; and microvesicles or exosomes for mammalian cells [10–14]. In this regard, microbiota-derived EVs have been identified as a carrier in host-bacteria interplays that, in terms of immune receptors, cause immune reactions [15]. It has been documented that non-pathogenic and pathogenic Gram-negative bacteria can generate vesicles [16]. The analysis and characterization of OMVs indicate that bacterial pathogens generate these secretory components to translocate virulent ingredients such as toxins, adhesins, and immunomodulatory factors, leading to cytotoxicity and modulation of immune response [16].

The ability of microbiota-derived OMVs to attach, enter, and deliver the cargos into host cells is based on the fusion capability of these vesicles to various membranes [17]. Based on the physiological characteristics of OMVs, the delivery of therapeutic cargos, such as microRNAs and proteins to tissues, has now been identified [18–21]. Also, bioengineering to target particular delivery of therapeutics has been administered by designing powerful nanocarriers [17, 18, 22]. The encapsulation, amphipathic nature, and bilayer topology of OMVs result in increased life span, enhanced stability, diminished side effects of these modules [22, 23]. Studies demonstrated that loading chemotherapeutic agents on OMVs, such as doxorubicin, can lead to increased accumulation of drugs in tumors and diminished toxicity compared to free doxorubicin [24, 25]. Besides, since MVs can easily transport molecules in the biological systems, they could be used to manufacture vaccines for effective antigen delivery [26]. For instance, it has been found that OMVs have powerful potential for adjuvants and are currently used in some vaccine platforms [27]. The essential activity of bacterial OMVs is to transfer biomolecules to particular targets [28]. Accordingly, they could be served as a new drug delivery tool because of various advantages, such as targeted delivery without causing toxicity on surrounding cells/tissue [28]. Bacteria OMVs can be loaded with many ligands using genetically handling their bacterial producers. These targeting ligands induce the deposition of drugs in target sites [28]. Besides, the OMV size is another advantage that allows the passively delivery of drugs to tumors via enhanced permeability and retention (EPR) inducing local immunity [28]. Targeted delivery to specific cells is another advantage of OMVs in drug delivery. OMVs originate from microorganisms and contain various pathogen-associated molecular patterns (PAMPs) that target cells as neutrophils and macrophages to quickly recognize and internalize [28]. Adjuvants can be highly beneficial in incorporated into OMVs, as they render full immunity and show low toxicity; hence, these molecules could also be employed as a novel mucosal delivery tool in vaccines [27]. In this review, we will discuss current updates on microbiota-derived OMVs in bacteria and their role in the host communication. We will also provide an overview of the current application and future perspective of OMVs for diagnostic and therapeutic purposes (Table 1).

**Table 1:** Outer membrane vesicles, bacterial producers, and their activity

Outer membrane vesicle-bacterium	Method for OMV isolation	OMV cargo	Function	References
A mutant *Escherichia coli* strain	Sucrose gradient ultracentrifuge	Delivering small interfering RNA (siRNA) targeting kinesin spindle protein (KSP)	OMVs can be used as cell-specific drug-delivery vehicles toward several cancers	[199]
*Bacteroides thetaiotaomicron, Bacteroides vulgatus, Bacteroides uniformis, E. coli, Barnesiella intestinihominis, Faecalibacterium prausnitzii, Bifidobacterium longum Parabacteroides distasonis,, Eubacterium rectale, Roseburia inulinivorans, Lactobacillus reuteri*	Membrane filtration and multiple cycles of ultracentrifugation	Proteins	OMVs provide great information in the communication between the microbiota and the host for preventing cancer and disease development	[200]
*E. coli* ΔmsbB OMVs	Filtration and ultracentrifugation	OMV encoding lipid A acyltransferase (msbB, had been inactivated (*E. coli* msbB−/−, ΔmsbB))	OMVs as therapeutic factors to treat various cancer through immunotherapy	[189]
*Pseudomonas. aeruginosa*	Filtration and ultracentrifugation	sRNA52320	sRNA52320 was plentiful in OMVs that diminished the LPS- and OMV-stimulated IL-8 generation. Besides, sRNA52320 attenuated OMV-stimulated keratinocytes-derived chemokine (KC) production and neutrophil infiltration	[54]
*Helicobacter pylori*	Filtration and centrifugation	sncRNAs (sR-2509025 and sR-989262)	Diminished LPS or OMV- stimulated IL-8 generation by cultured AGS cells (a human gastric adenocarcinoma cell line). Overall, these results are in accord with the notion that sncRNAs in OMVs derived from *H. pylori* have a new action in the pathogen-host interactions	[201]
*B. fragilis* and *B. thetaiotaomicron*	Tris ethylenediaminetetraacetic acid (EDTA)-sodium deoxycholate buffers/filtration, and multiple centrifugations	Different protein profiles and a safe endotoxin content	OMVs can be used in vivo studies as new therapeutic candidates	[194]
Meningococcal strain	Fractionated centrifugation	MenBvac, and MeNZB	Administration of the single and the combination of MenBvac and MeNZB vaccines considerably influence the outcomes of serogroup B meningococcal disease	[202]
ClearColi™, an endotoxin-free strain of *E. coli*	Ammonium sulfate, ultrafiltration, ultracentrifugation, and precipitation-based exosome isolation kit	Proteins	OMVs from the pre-stationary phase using ammonium sulfate (70%) + ultracentrifugation with enhanced yield could be used in vaccine studies	[203]
*Neisseria gonorrhoeae*	Filtration and ultracentrifugation	Naturally released OMVs (nOMVs) (rmp-deficient GC nOMVs)	These methods shedlight on future in vivo experiments on the anti-N's protective efficacy*. gonorrhoeae* stimulated by these nOMVs	[204]

*siRNA* small interfering RNA, *KSP* kinesin spindle protein, *UC* ultracentrifugation, *msbB* gene encoding lipid A acyltransferase, *sRNAs* short RNAs, *IL-8* interleukin 8, *sncRNAs* small noncoding RNAs, *EDTA* Ethylenediaminetetraacetic Acid, *AS* ammonium sulfate, *UF* ultrafiltration, *nOMVs* naturally released OMVs

## Extracellular vesicles

EVs are lipid-based vesicles containing lipids, proteins, and nucleic acids that are generated by various cells released into the surrounding milieu [29–31]. These vesicles are lipid packages and include exosomes, microvesicles, ectosomes, oncosomes, and apoptotic bodies [32]. EVs have different sizes (< 50 nm to several μm), chemical ingredients, and activities [33]. Besides, both commensal and pathogenic bacteria generate EVs categorized as OMVs produced by Gram negative bacteria or as MVs synthesized by Gram-positive bacteria [34]. Bacteria-derived EVs could influence host immunity, resulting in pro-inflammatory reactions [34]. On the other hand, probiotic-derived EVs usually cause immune modulation [34]. In this section, we will discuss and provide an overview of the latest information on EVs derived from the host and bacteria.

### Host-derived extracellular vesicles

In the host, micro-vesicles (MVs), exosomes, and apoptotic-derived bodies are listed to characterize host-derived EVs based on their biogenesis profile through membrane shedding, multicellular bodies, and apoptosis [35]. MVs are plasma membrane-derived vesicles with a size range of 100–1000 nm and are generated by vesiculation from eukaryotic cells. In this process, the asymmetry of phospholipid membrane mediated by cytoskeletal remodeling and enhanced cytosolic calcium play a vital role in shaping MVs [36]. MVs differ from other EVs in terms of the contents of phospholipids and proteins on their surface [37]. The importance of MVs in the propagation of coagulation and platelet aggregation due to the activity of membrane phospholipids has been addressed [38, 39]. Also, a growing body of evidence exhibits a connection between the overproduction of MVs and inflammatory reactions due to enhanced MV formation following the induction of tumor necrosis factor (TNF) [40].

Exosomes, another type of EVs, are sphingolipid- and cholesterol-rich membranes with a size range between 30 and 150 nm generated in all host cells [41]. It has been noted that exosomes are synthesized through inward budding of the endosomal compartments, followed by the fusion of multi-vesicular bodies to the cell membrane and the generation of intraluminal vesicles into the extracellular milieu [36]. It is known that the cargo of exosomes includes proteins, metabolites, lipids, as well as nucleic acids (mRNA, miRNA, and DNA) [36]. Exosomes can interact or be generated and internalized by recipient cells through various mechanisms such as fusion to the plasma membrane and/or adhesion to receptors mediating endocytosis [42, 43]. Finally, apoptotic bodies, another type of host EV, are larger than exosomes and contain cellular organelles, nuclear materials, and membrane/cytosolic contents. They are produced during the late phase of apoptosis [44]. Also, apoptotic bodies expose phosphatidylserine in their outer leaflet [44].

### Microbiota-derived extracellular vesicles

Like other organisms, bacteria generate EVs with a size < 300 nm as a communication tool [45]. Bacteria-derived EVs could cause a particular advantage via the horizontal transfer of resistance genes to other bacteria [46]. Also, these vesicles are a detoxification system that facilitates the depletion of toxic materials from mother bacteria [46]. Besides, bacteria-derived EVs prompt their adaptation to a new condition, as seen in commensal bacteria in which their EVs are involved in the colonization of the gastrointestinal tract [47]. Most Gram-negative-derived EVs are categorized as OMVs, a bleb form of OM that contains lipids, lipoproteins, and OM proteins [48]. Also, several Gram-negative bacteria could produce another type of EV, outer-inner-MVs, containing cytoplasmic and periplasmic components such as adenosine triphosphate (ATP), DNA cytoplasmic proteins [49].

Some conditions are necessary for vesiculating and synthesizing bacteria-derived vesicles [50]. Studies performed on vesiculation mutants have found that vesiculation does not stem from lysis or disintegration of the bacterial envelope [51]. In summary, it has been found that survival is the main advantage of vesicle formation in bacteria, causing the liberation of toxic material and misfolded proteins and/or eliminating the surface-attacking factors involved in micro-nutrient acquisition [51].

## Outer membrane vesicles characterization and biogenesis

**Fig. 1:**
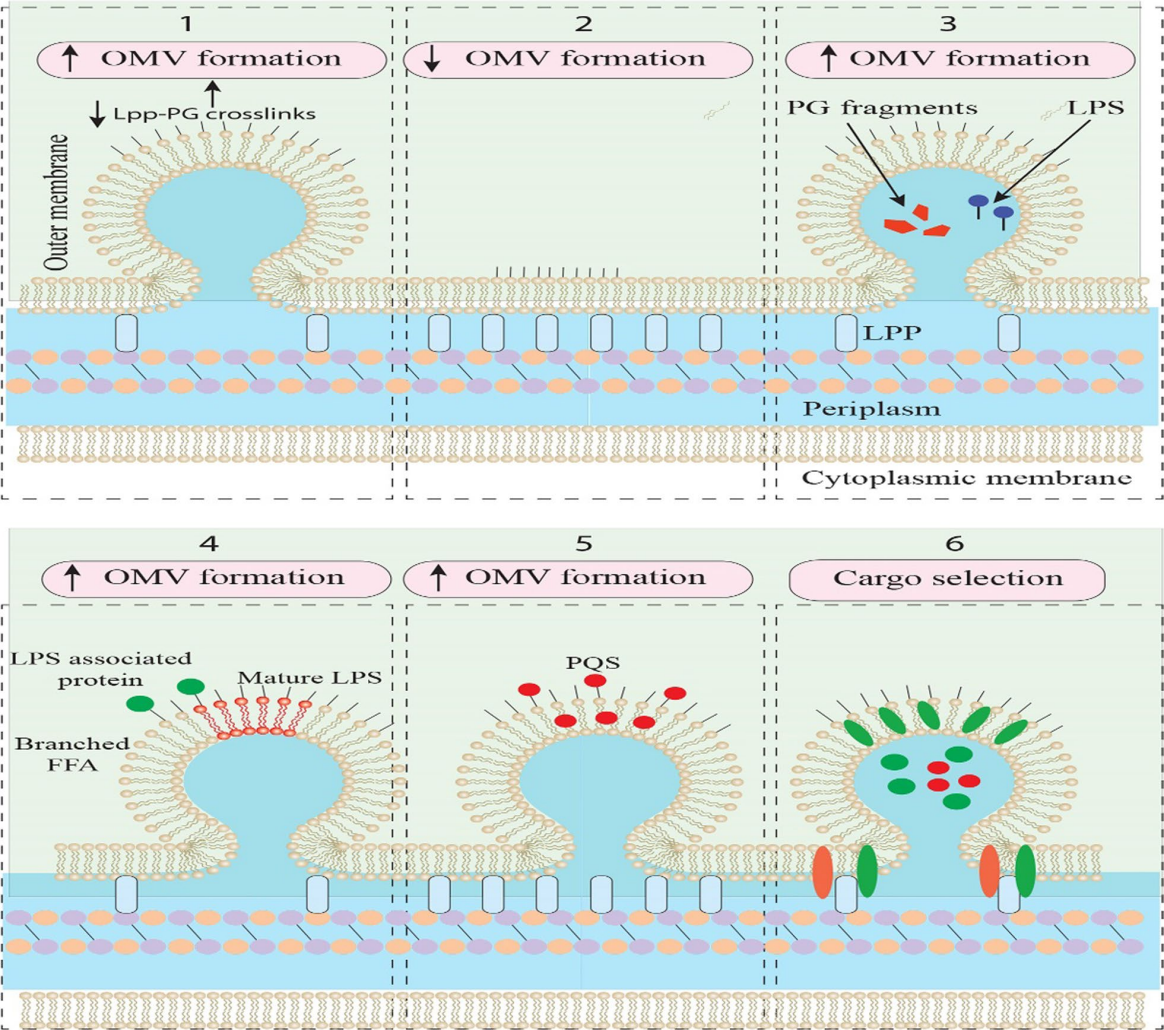
Biogenesis and cargo of outer membrane vesicles. It has been found that some components impact the OMV biogenesis including (1) Peptidoglycan endopeptidases, (2) cross-links of Meso-diaminopimelic acid– Meso-diaminopimelic acid within the peptidoglycan, (3) LPS or peptidoglycan fragments, (4) LPS-associated molecules, (5) insertion of PQS into the outer leaflet of the outer membrane, and (6) envelope components. OMV, outer membrane vesicle; LPS, lipopolysaccharide, PQS, Pseudomonas quinolone signal

Gram-negative bacteria-derived OMV, a bilayer spherical nanostructure (100–300 nm) with an internal cavity created into the extracellular milieu, made of the phospholipid bilayer, lipopolysaccharide (LPS), membrane protein, cell wall components, peptidoglycan, ion metabolites, signaling molecules, and nucleic acids (Fig. 1) [52–54]. Bacterial pathogen-derived OMVs are enriched with proteins involved in an invasive activity that causes efficient internalization of these vesicles into host cells [18]. Invasins, and type III secretion system-dependent integration of the hydrophobic proteins IpaD, IpaB, and IpaC (key virulence factors) of *Shigella flexneri* and the Ail protein of *Escherichia coli* is considered exemplary proteins, facilitating the process of internalization [55]. Gram-negative species include *E. coli*, *Shigella sp.*, *Pseudomonas aeruginosa, Campylobacter jejuni*, *Salmonella sp.*, *Helicobacter pylori*, *Vibrio sp.*, *Neisseria sp.*, and *Borrelia burgdorferi*, have been found to generate OMVs [50, 53, 56–67]. Besides their communication activity, Gram-negative-derived OMVs can transfer bacterial virulence factors as cargos to OMVs, leading to increased bacterial survival [68, 69].

To produce OMVs, OM must be released from the underlying peptidoglycan and swell outwards so that the vesicle membrane can detach [50]. Besides, the biophysical attribute of the OM-lipids and their interplays with proteins or other components that impact membrane bending has crucial activity in the biogenesis of OMVs [50]. Multiple models of OMV biogenesis have been proposed [18]. Studies found that reducing the cross-linking bond between OM and peptidoglycan induces the formation of OMVs [70, 71]. Vfgl, a different bacterial lipoprotein that iscontribute to the peptidoglycan production and degradation and mediates OMV biogenesis in *Adherent-invasive E. coli (AIEC)* and *E. coli* K12 strains [72]. These properties are presumably mediated by enhancing the synthesis of peptidoglycan or downregulation of lytic transglycosylases, leading to the maintenance of turgor pressure on the membrane [72, 73]. An increase in the number of OMVs produced as blebs to OM relieves the cells from the turgor pressure caused by peptidoglycan and muramic acid during cell wall synthesis [18].

In a study conducted by Mashburn and Whiteley [74], they found that enrichment of OM with phospholipids and LPS leads to the production of OMV. Besides, it has been shown that membrane curvature transformations via the membrane insertion of PQS (2-heptyl-3-hydroxy-4-quinolone), a quorum-sensing (QS) molecule, cause the formation of OMV in *P. aeruginosa* [75, 76]. Also, sequestration of positively-charged components and destabilization of calcium (Ca^2+^) and magnesium (Mg^2+^) by PQS can enhance the anionic repulsion of LPS, resulting in OMV formation [74]. Increased generation of OMVs has been detected by adding chelating agents, such as ethylenediaminetetraacetic acid (EDTA) [74, 77]. Also, OM proteins such as TolA/B (Tol-Pal), outer membrane protein A (OmpA), YbgF, and LppAB (all stabilize OM by increasing protein–protein or protein-peptidoglycan interplays) participate in the biogenesis of OMVs [78]. Some stress factors, such as high temperature and antibiotics also promote the production of OMVs [17, 79].

## Role of outer membrane vesicles in bacteria

The pathogenic role of Gram-negative bacteria OMVs in infection has been well documented; nevertheless, the advantages of OMVs for non-pathogenic microorganisms are still under investigation [50]. The formation of OMVs gives bacteria advantages, although the energy cost needed to produce these large macromolecules would be high [50]. OMVs mediate the transfer of DNA fragments, cytotoxins, autolysins, and virulence factors [80–82]. The generation of OMVs helps bacteria communicate and interact with host cells [18]. OMV, among its unique activity in diverse physiological and pathological functions, has been found to play a pivotal role in acquiring micro-nutrients, stress reactions, and translocation of adhesion, toxins, and virulence components to evade the host immune reactions [18].

Interestingly, the diversity in peptidoglycan structure makes bacteria prone to death by OMVs, and the cytotoxicity of OMVs would be outstanding for those bacteria possessing the same peptidoglycan structure [18, 83]. The neutralization of some bacteria; activity is compromised because of the same degradative enzymes in bacteria and OMVs, resulting in less susceptibility to degradation [18]. The fusion of OMVs to a non-self-strain enhances the susceptibility of bacteria to degradative enzyme systems [84]. The enzyme cargo of OMVs enables bacteria to distinguish between self and non-self-cells, resulting in the target-specific eradication of non-similar cells [85]. For instance, OMVs derived from this system are operational in a Gram-negative bacterium, *Lysobacter sp.,* that generates endopeptidase L5, resulting in degrading other competing Gram-negative bacteria [85]. Also, the same system for peptidoglycan hydrolase and OMVs containing peptidoglycan hydrolase produces destruction effects after making a clear distinction for non-self-microorganisms [79, 86, 87].

The packaging of enzymes, such as glycosidases and proteases, as cargo for bacteria-derived OMVs, shows an outstanding activity in acquiring micro-nutrients for microbial communities [18]. *Myxococcus Xanthus*-derived OMVs carry alkaline phosphatase that influences competitive bacteria, resulting in phosphate release that enhances the expansion of the multicellular community [88, 89]. Phosphoenolpyruvate (2-phosphoenolpyruvate, PEP), a catalytic cargo of OMVs carrying enolase, converts plasminogen into plasmin [18]. Also, PEP causes colonization of bacteria in the host to the degradation of matrix metalloproteins [90].

Additionally, it has been found that the limitation of metal ions in bacterial environments leads to competition between inter- and intra-species bacteria [17]. In this regard, loading trace elements on OMVs and serving them as a reservoir in interspecies competition result in the availability of metal ions for easy disposal through bacterial utilization [18]. Besides, the mutation in the stress-reactive genes enhances the formation of OMVs; also, the exposure of bacteria to antibacterial components has enormously evolved the production of these molecules, either by efflux pumps and/or the catalyzing the degradability of OMV cargo using the sequestration of antibacterial components from the extracellular environment [18, 91, 92]. It has been shown that the increased formation of surface receptors and ATP-binding cassette (ABC) transporters in OMVs, which act as sensors for micro-nutrients and transporters, can enhance bacterial survival [18].

Besides, it has been found that releasing exopolysaccharides via OMVs enhances the co-accumulation of bacterial cells in the biofilm mode of growth [93]. Biofilm is a surface adhering community of bacteria in response to stress that contains lipids, polysaccharides, proteins, nucleic acids, and appendages such as pili, flagella, as well as OMVs [5, 93–97]. The conversion from a planktonic growth mode into a biofilm mode of growth protects bacteria from numerous stress situations, such as starvation, desiccation, and anti-bacterial drugs [98]. In a biofilm, OMVs give a survival advantage to bacteria because it renders drug resistance with the help of biofilms that protect the embedded bacterial cells from anti-bacterial agents [99]. The connection of OMVs to the *P. aeruginosa* biofilm has intimidated the relation between stress and the increase of OMV formation during stress conditions [98, 99].

The interplay between bacteria with their host cells stimulates the generation of OMVs carrying different cargos, such as outer surface protein (Osp) A and OspB in *B. burgdorferi*, and BabA, SabA, and VacA in *H. pylori*, and UspA1 in *Moraxella catarrhalis* and aminopeptidase in *P. aeruginosa* [79]. GN-derived OMVs act as a bridge to enhance the bacterial adhesion to the host tissues and are also employed to increase bacterial adherence to the epithelial linings of the intestine and respiratory tract, leading to failure in bacterial elimination [18].

## Role of outer membrane vesicles in host

Despite the unraveled mechanism underlying OMV biogenesis, the effect of bacterial OMVs, particularly on host cells, is a matter of numerous studies. OMVs can bypass the epithelial cell barrier and enter host cells [100]. Subsequently, OMVs will be presented by immune cells, such as macrophages (MQ), neutrophils, and dendritic cells (DCs) in the submucosa and mediate inflammatory reactions against OMVs [48, 100, 101]. Besides, adaptive immune cells, including T and B lymphocyte cells, will be triggered by signal molecules produced in response to antigen-presenting cells [65, 66, 100].

OMVs, in combination with PAMPs, such as porins and LPS, induces powerful immune reactions in endothelial cells and stimulate the pattern-recognizing receptors (PRRs) on MQ cells [68, 102]. It has been found that OMVs mediated by toxins, such as cytolysin A (ClyA), leukotoxin, and LPS, are more potent than their soluble forms [16, 103]. For instance, the release of stx1 and stx2 of *Shigella dysenteriae* and Shiga toxin of enterohemorrhagic *E. coli* (*EHEC*) O157:H7 as cargo for OMVs efficiently suppress the process of protein synthesis in the host [104, 105]. GN-OMVs harbor many virulence components, including LPS, cystic fibrosis transmembrane conductance regulator (CFTR) inhibitory factor (Cif), hemolytic phospholipase C (plcH), and alkaline phosphatase, and they remarkably influence the host cells [106]. Toxins and virulence factors formation help bacterial cells invade the host, hijack host machinery to acquire micronutrients, and suppress host immune reactions that are fundamental for survival in the host [18].

Some studies showed that OMVs could cause phenotypic alterations in host cells [107, 108] along with inflammatory reactions when exposed to the host cells [100]. For example, OMVs of *Stenotrophomonas maltophili* stimulate powerful inflammatory responses in A549 cells (lung epithelial cells) [109]. OMVs of *V. cholerae* trigger inflammatory mediators by synthesizing active proteases [110].

Additionally, OMVs belonging to *P. aeruginosa* stimulate inflammasome formation via caspase-5 in THP-1 monocyte cells [111]. It has been shown that OMVs isolated from *E. coli* incite immune reactions and induce the expression of interleukin-8 (IL-8) in intestinal epithelial cells [112, 113]. Nevertheless, such interplays would be different between various bacterial OMVs. In this regard, OMVs of *Acinetobacter baumannii* have been indicated to possess hemolytic, phospholipase, and leucotoxic effects on blood cells [114]. Besides, OMVs derived from *H. pylori* exhibit a crucial activity on the degranulation of eosinophil cells [115]. OMVs of *Aggregatibacter actinomycetemcomitans* can be internalized in embryonic kidney cells and induce innate immune reactions [116]. OMVs of *Porphyromonas gingivalis* stimulates calcification of vascular smooth muscles via Extracellular Signal-regulated Kinase 1 and 2 (ERK1/2)–Runt-related transcription factor 2 (RUNX2) and induce innate immune reactions by endothelial cells [117, 118]. OMVs of probiotic *E. coli* reinforce the epithelial barrier via the modulation of tight-junctions (TJ) expression in intestinal cells [119]. OMVs are capable of enhancing the expression of cell adhesions, as employed by *E. coli* to increase the binding of the bacterium to endothelial cells [120]. Finally, OMVs derived from *Campylobacter jejuni* play proteolytic effects on the cleavage of E-cadherin and Occludin proteins expressing on intestinal epithelial cells [121].

Of note, it has been found that bacteria-derived OMVs affect the activity of host immune cells [100]. For instance, OMVs can stimulate the production of inflammatory cytokines by neutrophils [100]. OMVs isolated from *Neisseria meningitides* can activate neutrophils to release pro-inflammatory cytokines and chemokines, such as IL-8, interleukin1-β (IL1-β), TNF-alpha (TNF-α), macrophage inflammatory protein 1α and 1β (MIP-1α and MIP-1β) [122]. Also, it has been found that interferon-gamma (IFN-γ) could alter the level of these cytokines to preserve the chronic inflammation situations [122]. It shows that OMVs could involve in protective immunity toward infection and these reactions to OMVs are similar to those exerted by bacterial infection [100]. Additionally, some virulence factors transferred by OMVs could oppress the antibacterial activity of neutrophils and hence involve in the attenuation of cytokine generation [100]. OMVs belonging to *Uropathogenic E. coli (UPEC)* can transfer cytotoxic necrotizing factor type 1 (CNF1), a bacterial toxin, which diminishes the membrane fluidity and causes functional impairment in neutrophils, resulting in decreased activity of cytokines and chemokines [100, 123]. Despite the impact of OMVs on neutrophils, recent findings demonstrate that OMVs isolated from *N. meningitides* could be neutralized by plasma and bactericidal/permeability-increasing protein (BPI), which is an essential protein found in the azurophilic granules of neutrophils [124]. It has been found that when neutrophils prevent bacterial invasion, in some cases, these innate immune cells degrade themselves to induce a defense mechanism toward bacteria [100]. Neutrophil extracellular trap (NET) is a killing factor that enables neutrophil cells to stop bacterial pathogens [125]. Most importantly, it has been noted that bacteria-derived OMVs can activate the formation of NETs [126]. Nevertheless, in terms of *N. meningitides,* this pathogen could escape NETs, enhancing the OMVs levels and the progression of infection [126].

Bacteria-derived OMVs could stimulate DCs by co-stimulatory molecules and cytokine expression [127]. *N. meningitides*-derived OMVs activate DCs by the expression of accessory molecules (CD40, CD83, CD80, and CD86), human leukocyte antigen (HLA)-DR, and programmed death-ligand 1(PD-L1) [100]. Besides, DCs activated by *N. meningitides-*derived OMVs generate cytokines, such as IL-1β and Interleukin 6 (IL-6) [128]. OMVs derived from *H. pylori* activate DCs to produce hemeoxygenase-1 (HO-1) through activating protein kinase B (PKB) (also known as Akt)- Nuclear factor erythroid 2-related factor 2 (Nrf2) and mammalian target of rapamycin (mTOR)-κB Kinase- Nuclear factor-κB (NF-κB) pathways [129]. In summary, the exposure of DCs to bacterial OMVs can stimulate innate immune reactions toward infection [100].

Macrophages could elicit powerful immune reactions when exposed to microbiota-derived OMVs [100]. OMVs stimulate macrophages to generate pro-inflammatory cytokines [100]. The pretreatment of macrophages with OMVs leads to evoked inflammatory responses [80, 130, 131]. It has been documented that bacterial OMVs phagocytosed by macrophages can induce the formation of IL-1β, TNF-α, and IL-8 via the activation of NF-κB [132]. Macrophages activated by OMVs derived from *P. gingivalis* produce IL-6, TNFα, Interleukin 10 (IL-10), Interleukin-12, p70 (IL-12 p70), IFN-β, and nitric oxide (NO) [133]. Also, OMVs of *Legionella pneumophila* initiate pro-inflammatory reactions in macrophages via toll-like receptor-2 and -4 (TLR2 and TLR4) pathways [134]. Meanwhile, OMVs enhance the replication of *L. pneumophila* inside macrophages, and it may characterize how OMVs increase the dissemination of *L. pneumophila* in the host cells [134, 135]. Guanylate-binding proteins are found as regulators of inflammation caused by OMVs derived from *E. coli* that could infect bone marrow-derived macrophages [136]. In addition, it has been shown that macrophages activated by OMVs can cause adaptive immune reactions [100]. In this regard, OMVs isolated from *N. meningitidis* and *K. pneumoniae* trigger the expression of CD80, CD86, major histocompatibility complex-II (MHC-II), HLA-DR, and intercellular adhesion molecules-1(ICAM-1) molecules that support antigen presentation on the surface of macrophages [80, 137, 138]. Macrophages, antigen-presenting cells, activate T lymphocytes to detect antigens of OMVs and subsequently enhance adaptive reactions [139]. Notably, naive macrophages exposed to OMV of *Shigella boydii* can induce the polarization of CD4+T cells to T helper type 1 (Th1) [140]. Several studies show that microbiota-derived OMVs can change the metabolic remodeling of macrophages and stimulate apoptosis and pyroptosis [133, 141]. These phenomena can result in diminished levels and dysfunction of protective immune cells, which can be considered significant in disorder progression.

On the other hand, bacteria-derived OMVs play anti-inflammatory roles in infected host cells [100]. It has been found that macrophages exposed to OMVs can synthesize IL-10 [133, 140]. For example, OMVs belonging to *H. pylori* promote the formation of IL-10, an immunosuppressive cytokine, in peripheral blood mononuclear cells (PBMCs) and inhibit apoptosis in Jurkat T cells (JTCs) [142]. Therefore, it seems that these vesicles are a double-edged sword, as they exert immunostimulatory activity against infection and also, at the same time, facilitate bacterial production by limiting immune cells to attack bacteria.

When bacteria-derived OMVs enter the host cells, antigen-presenting cells present their cargo antigen toward CD4+T lymphocytes and induce differentiation of T-helper cells toward Th1, Th2, and Th17 cells involved in cellular and humoral immune reactions [100]. OMVs have powerful adjuvant influences on cross-priming and contribute to developing CD4+ and CD8+T cells [143]. Nevertheless, it has been demonstrated that OMVs can inhibit T response and growth [143]. *N. meningitides*-derived OMVs transfer opacity-associated protein (Opa) that can influence the proliferation of T lymphocytes by changing receptor binding [144]. OMVs of *H. pylori* are reported to suppress the proliferation of T lymphocytes by stimulating Cyclooxygenase-2 (COX-2) in monocyte cells [145]. Besides, transferring of Porin B (PorB) by OMVs of *Neisseria gonorrhoeae* could inhibit the proliferation of CD4+T lymphocytes, while PorB proteosomes alter immunosuppressive reactions [146].

B-lymphocytes participate in humoral immunity through antibody synthesis to defend the host against microbial pathogens, and these cells need T lymphocytes to react to microbial antigens [100]. OMVs of *Salmonella Typhimurium* stimulate priming of B and T lymphocytes, and specific Immunoglobulin G could be recognized in *in-vivo* models immunized with OMVs [80]. It has been detected that OMVs can directly activate B lymphocytes [147]. In order to characterize OMVs-B cell interaction, a novel mechanism can explain the stimulation of B lymphocytes by OMVs.

## Current applications of microbiota-derived outer membrane vesicles

**Fig. 2:**
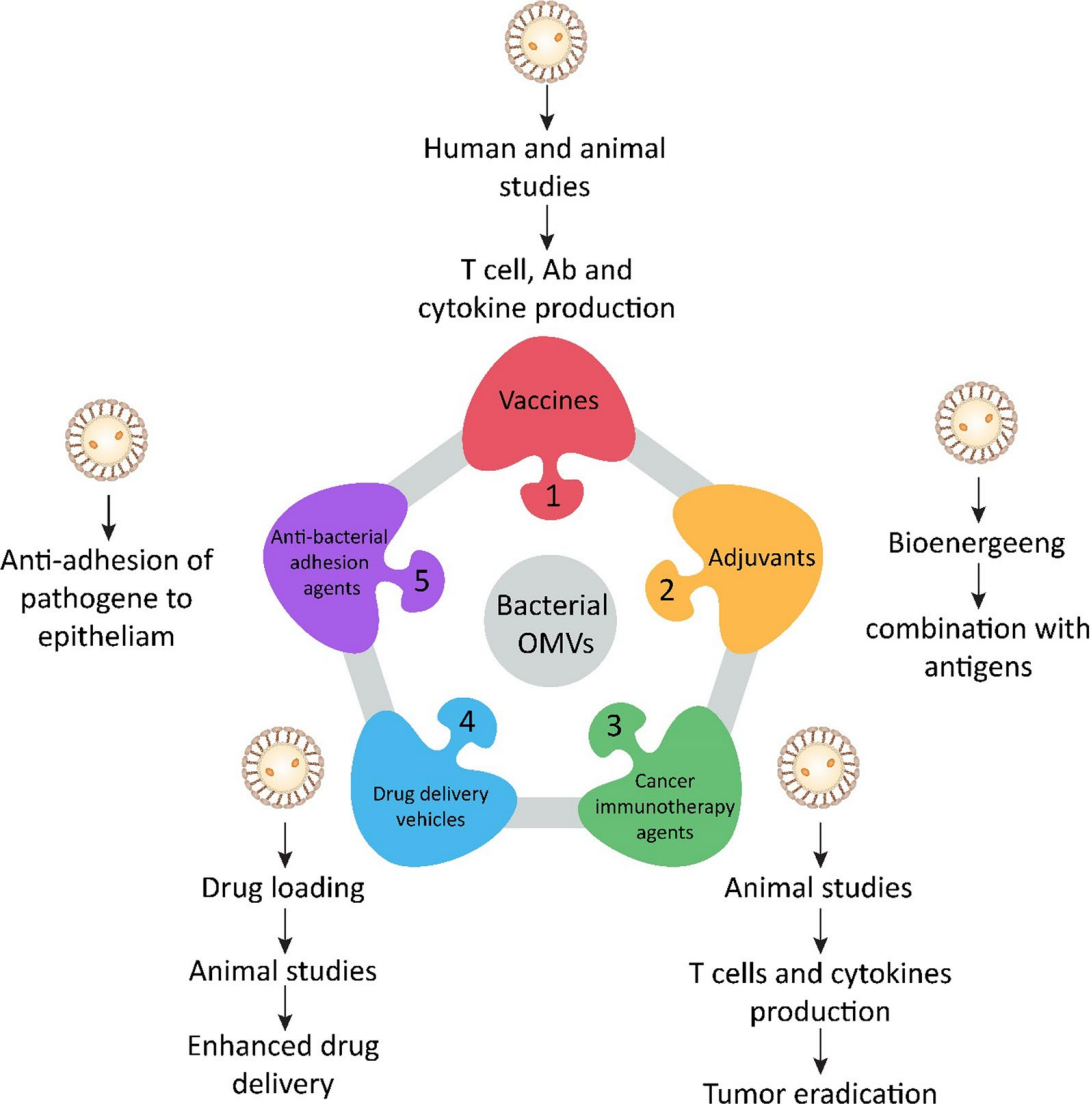
Biomedical applications of outer-membrane vesicles. (1) Vaccine, (2) adjuvant, (3) cancer immunotherapy agent, (4) delivery vehicle, and (5) inhibiting bacterial adhesion

Microbiota-derived OMVs possess different properties that make them attractive for various applications, such as drug delivery vehicles, microbial vaccines, cancer immunotherapy agents, adjuvants, and anti-bacterial adhesion components (Fig. 2) (Table 2) [28].

**Table 2:** Applications of outer membrane vesicles in medicine

Application	Method	Target	Description	References
*Vaccine*				
Meningococcal vaccine generating FetA	The OMV-MenPF-1 vaccine was formed by genetically modified *N. meningitidis* strain 44/76 to generate FetA	Human vaccine for broad protection toward MenB infection	As PorA and FetA are used as part of the usual surveillance of meningococcal disorders, changes mediated to invasive meningococcal disease can be used to reform PorA/FetA vaccine combinations to maintain optimal coverage	[205]
*N. gonorrhoea* vaccine	Odds ratios comparing disorders outcomes in vaccinated individuals versus unvaccinated individuals by multivariable logistic regression	Cases include incidences of gonorrhoea, chlamydia, and co-infection. As well as controls	This vaccine has found protection against gonorrhoea that provides a proof of principle that can inform prospective vaccine development for *N. gonorrhoea*	[206]
Meningococcal Vaccine (OMVs from *N. lactamica*)	The safety and immunogenicity of the vaccine *N. lactamica* OMV in the phase I clinical trial were evaluated	Ninety-seven healthy young adult male volunteers	Results showed this vaccine is safe and induces broad humoral immune reactions against *N. meningitidis*	[207]
Meningococcal vaccine (A hexavalent PorA OMV)	Using five wild-type P1.19,15 variants (A hexavalent PorA OMV vaccine), the serum bactericidal antibody (SBA) titers in pre-and post-vaccination in phases I and II trialswere evaluated	Toddlers and schoolchildren	These findings found implications for the use of PorA as a meningococcal serogroup B vaccine	[208]
Meningococcal vaccine	Native OMV (NOMV) vaccine prepared from a lpxL2(−) synX(−) mutant of strain 44/76 with opcA expression stabilized	Thirty-four volunteers	These results suggest that genetically modified NOMV vaccines can induce protection against group B meningococcus	[209]
*Adjuvant*				
OMV prepared from *N. lactamica* or *N. meningitides*	Mice were immunized with OMVs prepared from *N. meningitidis* and *N. lactamica* subcutaneously and intranasally	Mice	Results found that these OMVs are immunogenic when intranasally administered and act as effective intranasal adjuvants eliciting significantly increased IgA and IgG responses	[210]
Flagellin-deficient *Salmonella Typhimurium* OMVs	OMVs from flagellin-deficient *S. Typhimurium* and combined with outer membrane proteins from different Salmonella serotypes were purified and in vivo evaluated the response and cross-protection capacity to optimal vaccine composition	Mice	These OMVs induced significantly higher cellular immune reactions and displayed enhanced cross-protection for outer membrane proteins against wild-type virulent *Salmonella*	[211]
Penta acylated LPS-OMVs generated from ΔmsbB/ΔpagP mutant of *E. coli* W3110 (mOMV), and Hexa-acylated LPS-OMV generated from wild-type *E. coli*W3110 (wOMV)	T cell adjuvant activity of Penta acylated LPS-OMVs compared to Hexa-acylated LPS-OMVs. Penta-acylation of LPS renders mOMV less endotoxic than wOMV	Antigen-specific T cell priming in vitro and in vivo	It has been proposed that Penta acylated LPS-OMVs are a safe vaccine adjuvant for T cell priming and could further develop	[143]
*Drug carrier*				
Transformed *E. coli*-derived OMVs, detoxified by lysozymes	Mice were subcutaneously and intranasally immunized with OMVs from *N. meningitidis* and *N. lactamica*, as well as live cells	The carrier for transdermal drug delivery	This study shows that transformed *E. coli*-derived OMVs, detoxified by lysozymes, are promising nanoplatforms in tumor targeting and drug delivery with high efficacy and biosafety	[212]
Delivering small interfering RNA (siRNA) targeting kinesin spindle protein (KSP)	Bioengineered bacterial OMVs with low immunogenicity that can target and kill cancer cells in a cell-specific manner by delivering siRNA targeting KSP were described	Cancer cells	These OMVs had the potential as cell-specific drug-delivery vehicles to treat some cancers	[199]
*Cancer immunotherapy*				
Cancers	The potential of bacterial OMVs as therapeutic agents to treat cancer via immunotherapy was examined	Mice	Remarkable capability of bacterial OMVs to effectively induce long-term antitumor immune reactions that could eradicate established tumors without adverse effects. Moreover, these OMVs induce the production of antitumor cytokines interferon-gamma (IFN-γ) and C-X-C motif chemokine ligand 10 (CXCL10)	[189]
Cancers (Differentially packaged sncRNAs in *Helicobacter pylori* OMVs)	Differentially packaged sncRNAs in *H. pylori* OMVs were identified, and OMV sncRNAs to human gastric adenocarcinoma cells were transferred	Human gastric adenocarcinoma cells (host mRNA)	It has been found that sR-2509025 and sR-989262 as sncRNAs that interact with host cells through OMV secretion and reduce the secretion of Interleukin 8 (IL-8), which targets mRNAs encoding multiple kinases in the LPS-stimulated mitogen-activated protein kinase (MAPK) signaling pathway, have not been thoroughly elucidated	[201]
Bacterial OMVs	A nanovaccine using bacterial biomembranes as carriers for antitumor therapy was developed. In this regard, the strong adjuvant effect of OMVs was used to induce the anti- basic fibroblast growth factor (BFGF) autoantibodies. The whole BFGF molecule was loaded onto OMVs and used for tumor therapy	Tumor	The current study found that OMVs successfully induce the persistent anti-BFGF autoantibodies to inhibit tumor growth and metastasis	[213]
Bactrial OMVs	OMVs- mesoporous silica (MSN)- 5-fluorouracil (5FU) were prepared by high-pressure co-extrusion, and size, drug loading, thermal gravity analysis, cytotoxicity, and cell uptake were characterized	Colon cancer	The study provided a promising nano platform for the targeting treatment of colon cancer	[214]
*Biomarker*				
sRNA52320 in OMVs	RNA-Seq to characterize differentially packaged sRNAs in *Pseudomonas aeruginosa* OMVs was used, and transfer of OMV sRNAs to human airway cells was done	Human airway cells	These findings are consistent with the hypothesis that sRNA52320 in OMVs is a novel mechanism of host–pathogen interplays whereby some bacteria, such as *P. aeruginosa,* reduce the host immune reactions	[54]
Salivary OMV and DNA methylation of small extracellular vesicles	The healthy, gingivitis, and periodontitis groups were compared in terms of salivary extracellular vesicles in the CD9 + salivary extracellular vesicles subpopulation, Gram-negative bacteria-enriched LPS + OMVs, and global DNA methylation pattern of 5-methylcytosine (5mC), 5-hydroxymethylcytosine (5hmC), and N6-Methyladenosine (m6dA)	Healthy gingivitis and periodontitis individuals	The results show that global salivary extracellular vesicles methylation could be a potential biomarker for human periodontitis	[182]

*OMVs* outer membrane vesicles, *MenB* capsular group B *Neisseria meningitidis*, *SBA* serum bactericidal antibody, *OMP* outer membrane protein, *ELISA* enzyme-linked immunosorbent assay, *CFSE* carboxyfluorescein diacetate, *ICS* intracellular cytokine staining, *BMDCs* bone marrow-derived dendritic cells, *LPS* lipopolysaccharide

### OMV as a drug delivery system

As previously noted, the essential activity of bacterial OMVs is to transfer biomolecules to particular targets [28]. Accordingly, they could be served as a new drug delivery tool because of various advantages, such as targeted delivery without causing toxicity on surrounding cells/tissue [28]. Bacteria OMVs can be loaded with many ligands using genetically handling their bacterial producers. These targeting ligands induce the deposition of drugs in target sites [28]. Besides, the OMV size is another advantage that allows the passively delivery of drugs to tumors via EPR [28]. Targeted delivery to specific cells is another advantage of OMVs in drug delivery. OMVs originate from microorganisms and contain various PAMPs that target cells to recognize and internalize [27] quickly.

The loading of drugs on bacteria-derived OMVs can protect these drugs from denaturation and degradation before reaching the targets [28]. Most importantly, in the case of cancer therapy, OMVs stimulate immune reactions that can be useful for the better elimination of tumors [28]. Nevertheless, if the immune reactions are not correctly controlled, they can damage the host. This implies why detoxified OMVs with lower inflammatory response capability are warranted. Taken together, the administration of microbiota-derived OMVs as a delivery tool would be promising for drug delivery systems.

### OMVs as bacterial vaccines

Various models of vaccines are applied to protect the host from associated microbial infections [28]. As a result of possessing the pathogen components, vaccines can stimulate long-lasting pathogen-specific immune reactions [28]. Of note, microbiota-derived OMVs are currently noted to be used for this goal because OMVs contain some PAMPs, and also, they could enter the lymph nodes via lymphatic drainage after phagocytosis by antigen-presenting cells [28, 148]. The detection and uptake of bacteria-derived OMVs by antigen-presenting cells enhance their antigen presentation, co-stimulatory molecules formation, as well as pro-inflammatory cytokines formation [148].

One study showed a potential bacteria-derived OMV-based vaccine that was derived from *N. meningitides*. This type of OMV could be employed as an adjuvant to increase the immune response against meningitis type B [28]. OMV-derived vaccines have been used clinically for meningitis outbreaks in some countries, such as Norway and Cuba (efficacy up to 70%) [149–153]. This type of vaccine contains some antigens, such as PorA [154, 155]. The PorA protein is a crucial immunogenic factor of OMVs derived from *N. meningitides* and found in various strains [153]. Therefore, the immune reaction stimulated by OMV-based vaccines, similar to other types of vaccines, is specific to strain. Accordingly, a novel multivalent PorA vaccine has been administered from bioengineered OMVs containing various PorAs in the Netherlands [156, 157]. This OMV-based vaccine stimulated a four-fold enhancement in humoral immunity in a phase I trial [156, 157]. Other proteins in bacteria-derived OMVs also induce host reactions [153]. The FDA and European Medicines Agency approved the MenB vaccine. This vaccine contains OMV ingredients, such as minor proteins and PorA, to induce anti-pathogen reactions [28, 153]. Bacteria-derived OMV-based based vaccines have been extensively studied against bacterial pathogens, including *S. flexneri, H. pylori*, *V. cholera*, and *S. Typhimurium* [28, 158, 159]. It should be noted that these OMVs are generated from their parent bacteria that have been found to induce cellular and humoral immune reactions [28]. The generation of antibodies such as different Immunoglobulin G (IgG) and Immunoglobulin M (IgM) can be specific to pathogenic proteins as well as LPS [28]. In summary, different OMV vaccines with low toxicity and higher efficiency will be examined and entered the clinic.

### OMVs as adjuvants

It has been well-documented that immunization with classical vaccines containing proteins or other antigens stimulates a medium immune reaction, particularly for cellular reactions [160]. Hence, currently, adjuvants were further evaluated to increase and shape immune reactions toward a particular antigen. In this regard, adjuvants act via producing depot, enhancing antigen presentation and uptake to lymph, and directly stimulating immune responses [161]. Thus, adjuvants can diminish the number of antigens and doses to achieve therapeutic and prophylactic goals, reducing the cost of treatment. Some properties of OMVs include non-replicating ability when isolated from their bacterial origin, size of < 300 nm, and containing PAMPs [162]. These properties made them an ideal candidate to be utilized as adjuvants [48]. The non-replicating ability of OMVs, in contrast to their bacterial origin, can solve safety problems existing in the application of a completed form of bacteria. Also, the size of bacteria-derived OMVs facilitates their entry into different sites, such as lymph nodes via lymphatic drainage and also phagocytosis by antigen-presenting cells [162]. Also, the pathogen-like property of OMVs triggers their uptake by antigen-presenting cells [163, 164]. Various types of PAMPs present on OMVs can interact with PRRs expressing on antigen-presenting cells and induce their full activation, leading to powerful adaptive immune reactions [163, 164]. It has been reported that lipoproteins and LPS present on the membrane of OMVs interplay with TLR2 and TLR4 on the surface of antigen-presenting cells, enhancing the uptake and recognition of OMVs by these cells [165]. RNA and DNA cargo of OMVs can interact with TLR3 and TLR9 in endosomes, stimulating the proliferation of antigen-presenting cells [165]. The administration of adjuvants can stimulate the synergistic formation of cytokines by antigen-presenting cells, resulting in enhanced T lymphocyte and antibody formation [166, 167].

It should be noted that vesicular compositions of bacteria-derived OMVs facilitate the inclusion of various antigens [168]. Hence, the entry of these OMVs into antigen-presenting cells can also mimic these antigens and contribute to the presentation and processing of antigens [28]. Most importantly, OMVs can be engineered to produce antigens by genetic manipulation of their bacterial origin [28]. A novel OMV-based vaccine was recently designed by loading Poly-β-1,6-N-acetyl-D-glucosamine (PNAG), an immunogen generated by bacterial pathogens, on OMVs to cause a robust immune response against PNAG- bacteria [169]. It has been indicated that the treatment of mice with OMVs protected them against the lethal effect of various PNAG-forming bacteria [28]. Taken together, the potential of microbiota-derived OMVs as an adjuvant in developing novel vaccines would be of note.

### OMVs as cancer immunotherapy agents

The use of bacteria-derived OMVs for human cancer therapy is currently performed in multiple clinical trials [28]. The application of OMVs was relatively safer than live bacterial cells, as they are non-replicating particles [28]. OMVs contain different immunostimulatory components that help detect and uptake bacteria-derived OMVs and lead to the activation of immune reactions [28]. Due to the size of OMVs, they can enter or bind to tumor sites and stimulate local immunity via EPR effects [28]. In a study conducted by Kim et al. [170], they exhibited the remarkable anti-tumor activity of OMVs. They found that following the intravenous injection of OMVs are stored in tumor sites and stimulate anti-tumor immune reactions to eliminate tumors [170]. It has been shown that some OMV-derived bacteria can suppress tumor growth, and benefit cancer therapy [170]. Interestingly, the anti-tumor immune response stimulated by OMVs causes immunological memory in mice [170]. Notably, this anti-tumor influences the function of IFN-γ- and trypsin-sensitive proteins and has a crucial role in the formation of IFN-γ [170].

Bacteria-derived OMVs induce effective anti-tumor activity that can completely eliminate tumor sites and suppress tumor metastasis and recurrence [28]. Accordingly, a study by Chen et al. found that co-administration of bacteria-derived OMVs and chemotherapeutic drugs led to a better anti-tumor response. They loaded polyethylene glycol and the Arg-Gly-Asp peptide, a tumor-targeting ligand, on OMVs to enhance their blood circulation and enhance tumor-targeting properties [171]. In the next step, they coated OMVs with Tegafur, which made cancer cells sensitive to T lymphocytes and diminished the immunosuppressive cells such as myeloid-derived suppressor cells. These OMV-coated nanoparticles provided an anti-tumor activity that resulted in stimulating the host immune cells. The systemic injection of these OVMs increased the accumulation of particles in tumors via the EPR effect and active targeting through the Arg-Gly-Asp peptide [171].

### OMVs as diagnostic and therapeutic biomarker

A key function of bio-imaging methods is to aid in the early detection and management of diseases. OMVs can have exogenous bio-imaging probes created and fixed onto them to deliver a visual signal by optical, magnetic, or nuclear means [172]. Due to this property, research into the processes by which OMVs mediate bacterial-host communication can be conducted. According to this principle, OMVs could be detected in body fluids, and their molecular compositions reflect their origin; hence, OMVs can be considered novel prognostic and diagnostic biomarkers for many infectious diseases. OMVs possess some distinct advantages, such as the ability to act as noninvasive biomarkers generated by almost all pathogens, reflect the progress of the infection, show treatment response, protect their cargos during long-term storage, as well as the biodegradability in all body fluids [173].

DiR iodide, a lipophilic fluorescent dye, labels membranes. By identifying OMVs with DiR, Liu et al. [174] showed that *Akkermansia muciniphila* OMVs can infiltrate and aggregate in bone tissues to enhance osteogenic activity and prevent osteoclast formation. Non-covalently bound lipophilic fluorescent dyes are unstable and lose fluorescence quickly.

As previously mentioned, OMVs carry various bacterial components such as LPS, proteins, DNA, and RNA [48, 175]. Ghosal et al. [176] evaluated the extracellular component of *E. coli* and found that OMVs derived from the *E. coli* MG1655 strain contain small non-coding RNAs. Besides, Sjöström et al. [177] revealed that OMVs belonging to *V. cholerae* contain sRNAs. Also, Resch et al. [178] reported non-coding RNAs enriched in OMVs belonging to group A Streptococcus. Koeppen et al. [54] revealed an inter-kingdom regulation by sRNAs through bacterial OMVs in which sRNA52320 from OMV of *P. aeruginosa* could be transferred into epithelial cells in the lung and diminish the immune reactions induced by LPS via targeting IL-8 mRNA. These findings have promisingly noted secretory sRNAs' pathological and biological significance in OMVs.

Moreover, optoacoustic imaging can be done using bacterial vesicles. Melanin's extensive optical absorption makes it excellent for optoacoustic imaging[179]. Melanin can be spontaneously packed into OMVs by overexpressing tyrosinase in *E. coli*, a crucial enzyme in melanin formation. OMVs create an improved multi-spectral optoacoustic tomography signal and induce local warmth when irradiated [180]. Engineered OMVs can aggregate in mouse tumor tissue for imaging and photothermal treatment after systemic delivery. Polydopamine nanoparticles produced by oxidative polymerization of dopamine are melanin-like and can be incorporated into the OMV–cancer cell hybrid membrane for tumor-targeted photoacoustic imaging and photothermal treatment [9].

Several studies showed that Gram-negative periodontal pathogens, including *Treponema denticola*, *Tannerella forsythia*, *P. gingivalis*, *Fusobacterium nucleatum*, *Campylobacter rectus*, *Prevotella intermedia*, *Eikenella corrodens*, and *Peptostreptococcus anaerobius* that are mediated periodontal attachment and disorder progression can generate OMVs [181, 182]. It has been demonstrated that OMVs of *P. gingivalis* trigger bacterial co-aggregation and impact the bacterial structure in periodontal plaque via sub-gingival biofilm formation.[181, 182]. Hence, characterization and detection of saliva-specific bacteria-derived OMVs are crucial to many definitions of the microbiome–host interplays in periodontal disorders. Accordingly, Han et al. [182] evaluated the specific periodontal pathogen-derived OMVs in salivary from periodontitis patients. They found that 5mC hypermethylation in salivary OMVs could distinguish periodontitis individuals from healthy individuals [182]. This result shows that OMV methylation can be a promising biomarker for human periodontitis.

By interacting with intestinal epithelia and the mucosal immune system, commensal OMVs maintain intestinal homeostasis. *B. fragilis* OMVs prevent intestinal inflammation and colitis in mice [183]. *Bacteroides thetaiotaomicron* OMVs induce IL-10 expression in healthy colonic DCs but not in IBD patients [184]. *B. thetaiotaomicron*-derived OMVs modulate immunological responses, making them potential IBD therapies. OMVs can be combined with innate immunogenicity to improve immunotherapy effectiveness. OMVs can penetrate through the stratum corneum, making them suitable for melanoma treatment. Peng and Wang [185] developed *E. coli* producing TNF-related apoptosis inducing ligand (TRAIL) protein and modified OMVs with v3 integrin peptide, targeting ligand, and indocyanine green for melanoma treatment. Multifunctional OMVs can boost antitumor performance in cutaneous melanoma with transdermal photo-TRAIL therapy.

OMVs and their promising application as biomarkers are useful candidates for therapeutic approaches. Despite the challenges in the clinical administration of OMVs, their physiological and biological characteristics have great power as diagnostic and therapeutic tools. In summary, further research can help introduce potential biomarkers and facilitate the clinical application of bacteria-derived OMVs.

## Limitation of OMV application

Currently, considerable investigations have been carried out to evaluate the role of OMVs in bacterial communication and infection development [186, 187]. Besides, many groups have examined OMVs for their potential as delivery vehicles, bacterial vaccines, adjuvants cancer immunotherapy agents [22, 28, 188–190]. Nevertheless, there are some limitations, such as a lack of inadequate terminology, standardized methodology for the purification and/or isolation of different OMVs, and technical challenges in quantification and characterization [34, 191].

The difficult separation and purification processes necessary to get significant amounts of these microscopic vesicular structures are one of the primary challenges of investigating OMVs. The majority of investigations identify ultracentrifugation and ultrafiltration as techniques [192]. Notably, the isolation process can impact the shape and yield of OMVs, increase OMV aggregation, and/or collect lipoproteins and other undesirable cell debris. Therefore, the optimal OMV separation approach should deliver high OMV yields without compromising vesicles for further experimental investigations or biotechnology applications.

The generation of next-generation vaccinations has a lot of potential with OMV-based vaccines. There are still a lot of difficulties, including yields of OMVs after isolation and the composition, which affects immunogenicity and toxicity. OMVs are naturally advantageous to the bacterium, but they are not created in significant amounts during bacterial growth. However, there can be a very easy way to improve OMV yields [191]. According to research, OMV release rises in response to stress. Environmental stress, such as pressure, temperature, or nutrient depletion stress, is the least serious type of stress that bacteria can endure.

Along with the toxicity of wild-type LPS, bacteria-derived OMVs with several TLR antagonists occurring in OMVs such as lipoproteins, flagellin, and other OMPs can cause uncontrolled reactions such as excess inflammation [27]. Hence, OMV endotoxin components must be eliminated after isolation; for example, in *Neisseria*, the Factor H binding protein must be isolated from OMPs due to its cytotoxic nature [27]. Another challenge is that LPS-deficient OMVs usually show less immunogenicity than wild-type bacteria-derived OMVs. Hence, an optimal balance in the effective changes in LPS, such as low toxicity and high immunogenicity, is warranted.

Most importantly, if microbiota-derived OMVs are commercialized for the abovementioned applications, mass production should be considered [193, 194]. The mechanism underlying the production of OMVs is not fully understood, and hence consistent formation may be complex [193]. In this regard, during the Upstream Process of pre-culture of bacteria, another antifoam was needed for many scales up in the fermentation process. In contrast, a significant number of antifoams are not compatible with the generation processes of OMVs. Their surfactants may influence OMV function or even interfere with the integrity and purification of OMV [195, 196]. However, the use of antifoam is still considered a standard approach to inhibit excessive foaming due to required aeration at different densities [193]. Alternative approaches for mechanical foam breaking have been evaluated as part of the scale-up during the fermentation process [27].

Additionally, external components such as temperature and in rare cases, the absorption of phages, also influence OMV generation. Also, oxidative stress due to cysteine depletion in *N. meningitides* and/or sodium carbonate in *V. cholera* can affect the yield volume of the recombinant OMVs [27]. Hence, it is required to enhance mediated production technology and environmental situations.

The poor yield of OMVs, which are released spontaneously by bacteria but in very small numbers, together with the possibility of low levels of important protective antigens on their surface, are further barriers to their use as vaccines [197]. OMVs also contain endotoxins and deoxycholate extraction followed by differential centrifugation from the homogenized bacterial bulk can increase yield and decrease endotoxin levels; these are typically referred to as OMVs made using this technique detergent-extracted OMVs.

Lastly, several studies noted that LPS derivatives have a similar impact when compared with WT-LPS in vivo. These species-specific reactions can cause differences in the signaling and induction of TLRs [198]. Thus, this reaction highlighted the difficulty of in vivo analysis of the safety of microbiota-derived OMVs in humans. Hence, to improve the challenge of OMV applications, many human trials are needed to examine their biological effects.

## Concluding remarks and future perspective

All in all, the current evidence implies that the gut microbiota and its metabolites have a crucial role in human health and disease. The disruption of the gut microbiota (which is called dysbiosis) balance can disturb the host's energy metabolism and immunity, significantly impacting the development of numerous human disorders. Recent investigations propose that OMVs could perform a critical role in shaping immune responses, including homeostasis and acute inflammatory responses. Following dysbiosis of the gut microbiota during infection, the number and type of these OMVs may change so that these molecules can be employed as targets for diagnosis. Also, we can apply OMVs as antibacterial agents. In this review, the application of OMVs for medical purposes, such as cancer immunotherapy, OVM-based vaccines, and drug delivery, were broadly addressed.

It should be noted that several obstacles exist in the application of these molecules, such as low yield volume and toxic effects owing to possessing some cytotoxic components (e.g., LPS). In this regard, some approaches have been proposed, such as genetic manipulation to reduce endotoxicity. One solution that seems to be optimal for increasing yields of OMVs would be heat induction [191]. In conclusion, future studies should focus on using OMVs and solving these challenges to pave the way for applying these molecules in the clinic.

## Data Availability

Not applicable.

## Abbreviations

IBD: Inflammatory bowel disease
IBS: Irritable bowel syndrome
MS: Multiple sclerosis
GI: Gastrointestinal
MVs: Membrane vesicles
EVs: Extracellular vesicles
OMVs: Outer MVs
MVs: Micro-vesicles
TNF: Tumor necrosis factor
AIEC: Adherent-invasive *E. coli*
QS: Quorum-sensing
Ca^2+^: Calcium
Mg^2+^: Magnesium
EDTA: Ethylenediaminetetraacetic acid
OmpA: Outer membrane protein A
MQ: Macrophages
DCs: Dendritic cells
PNAG: Poly-β-1,6-*N*-acetyl-d-glucosamine
PAMPs: Pathogen-associated molecular patterns
PRRs: Pattern-recognizing receptors
EHEC: Enterohemorrhagic *E. coli*
IL-8: Interleukin-8
ERK1: Extracellular signal-regulated kinase
RUNX2: Runt related transcription factor 2
TJ: Tight-junctions
IL1-β: Interleukin1-β
IFN-γ: Interferon-gamma
UPEC: Uropathogenic *E. coli*
CNF1: Cytotoxic necrotizing factor type 1
NET: Neutrophil extracellular trap
HLA: Human leukocyte antigen
IL-6: Interleukin 6
HO-1: Hemeoxygenase-1
PKB: Protein kinase B
MTOR: Mammalian target of rapamycin
IL-10: Interleukin 10
NO: Nitric oxide
