# Correction: Emerging role of microbiota derived outer membrane vesicles to preventive, therapeutic and diagnostic proposes

**DOI:** 10.1186/s13027-023-00484-0

**Published:** 2023-02-02

**Authors:** Saba Jalalifar, Hassan Morovati Khamsi, Seyed Reza Hosseini‑Fard, Sajad Karampoor, Bahar Bajelan, Gholamreza Irajian, Rasoul Mirzaei

**Affiliations:** 1 Microbial Biotechnology Research Center, Iran University of Medical Sciences, Tehran, Iran; 2 Department of Microbiology, School of Medicine, Iran University of Medical Sciences, Tehran, Iran; 3 Department of Quality Control, Razi Vaccine and Serum Research Institute, Agricultural Research, Education and Extension Organization (AREEO), Karaj, Iran; 4 Department of Biochemistry, School of Medicine, Tehran University of Medical Sciences, Tehran, Iran; 5 Gastrointestinal and Liver Diseases Research Center, Iran University of Medical Sciences, Tehran, Iran; 6 School of Medicine, Alborz University of Medical Sciences, Karaj, Iran; 7 Venom and Biotherapeutics Molecules Lab, Medical Biotechnology Department, Biotechnology Research Center, Pasteur Institute of Iran, Tehran, Iran

**Correction to:** **Infectious Agents and Cancer (2023) 18:3** 10.1186/s13027-023-00480-4

Following publication of the original article [1], the authors identified a typesetting mistake in the order of affiliations: the original affiliation 6 (Microbial Biotechnology Research Center, Iran University of Medical Sciences) should have been re-ordered to affiliation 1 and the original affiliation 1 (Department of Microbiology, School of Medicine, Iran University of Medical Sciences) should have been re-ordered to affiliation 2. All other affiliation should have been re-ordered accordingly.

In addition, the authors identified a mistake in the presentation of the original affiliations 1 and 6 (re-ordered to affiliations 1 and 2): the ‘University of Medical Sciences’ should have been presented as ‘Iran University of Medical Sciences’.

The affiliation errors are corrected in the author list and affiliation list of this Correction article and the original article [1] has been updated.
